# Reassessing access to intensive care using an estimate of the population incidence of critical illness

**DOI:** 10.1186/s13054-018-2132-8

**Published:** 2018-08-20

**Authors:** Allan Garland, Kendiss Olafson, Clare D. Ramsey, Marina Yogendranc, Randall Fransoo

**Affiliations:** 1 Department of Internal Medicine, University of Manitoba, 820 Sherbrook Street, Winnipeg, MB R3A1R9 Canada; 2 Department of Community Health Sciences, University of Manitoba, Room S113, 750 Bannatyne Avenue, Winnipeg, MB R3E0W3 Canada; 3 Manitoba Centre for Health Policy, University of Manitoba, Room 408, 727 McDermot Avenue, Winnipeg, MB R3E3P5 Canada

**Keywords:** Critical illness, Intensive care units, Health care quality, access, and evaluation

## Abstract

**Background:**

The consistently observed male predominance of patients in intensive care units (ICUs) has raised concerns about gender-based disparities in ICU access. Comparing rates of ICU admission requires choosing a normalizing factor (denominator), and the denominator usually used to compare such rates between subpopulations is the size of those subpopulations. However, the appropriate denominator is the number of people whose medical condition warranted ICU care. We devised an estimate of the number of critically ill people in the general population, and used it to compare rates of ICU admission by gender and income.

**Methods:**

This population-based, retrospective analysis included all adults in the Canadian province of Manitoba, 2004–2015. We created an estimate for the number of critically ill people who warrant ICU care, and used it as the denominator to generate critical illness-normalized rates of ICU admission. These were compared to the usual population-normalized rates of ICU care.

**Results:**

Men outnumbered women in ICUs for all age groups; population-normalized male:female rate ratios significantly exceed 0 for every age group, ranging from 1.15 to 2.10. Using critical-illness normalized rates, this male predominance largely disappeared; critically ill men and women aged 45–74 years were admitted in equivalent proportions (critical-illness normalized rate ratios 0.96–1.01). While population-normalized rates of ICU care were higher in lower income strata (*p* < 0.001), the gradient for critical illness-based rates was reversed (*p* < 0.001).

**Conclusions:**

Across a 30-year adult age span, the male predominance of ICU patients was accounted for by higher estimated rates of critical illness among men. People in lower income strata had lower critical-illness normalized rates of ICU admission. Our methods highlight that correct inferences about access to healthcare require calculating rates using denominators appropriate for this purpose.

**Electronic supplementary material:**

The online version of this article (10.1186/s13054-018-2132-8) contains supplementary material, which is available to authorized users.

## Background

Disparities in health and healthcare delivery are ubiquitous [1–5]. Identifying and eliminating them has become a special focus of governments and international groups [2, 5, 6]. Studies showing that men outnumber women in ICUs throughout Europe [7–9] and North America [10–13] have raised concern about gender-related disparities in ICU access [14].

Identifying disparities in access to any healthcare interventions involves comparing rates of use between subgroups; that is, the quotient of the number of people who received the intervention to some normalizing factor (denominator). Although the most commonly used denominator is the size of the population, creating population-normalized rates, these rates can be misleading in assessing access to care [15, 16]. Instead, the most appropriate denominator is the number of people whose medical condition warranted such care [17]. For ICU care, the correct denominator is the number of people who developed critical illness and were suitable candidates for ICU admission, producing critical illness-normalized rates. If men had higher critical illness rates but equal ICU access compared to women, their population-normalized rate would be higher, while their critical illness-normalized rates would be the same.

A method to estimate rates of critical illness in the population has been lacking [17]. Here we develop such a method and use it to evaluate equality of access to ICUs in the Canadian province of Manitoba, by gender and socioeconomic status (SES).

## Methods

We used population-based data including hospital abstracts and vital statistics for all Manitobans, which reliably identify the existence and timing of ICU care [18, 19]. In 2016, Manitoba had a population of 1.34 million [20], covered by a single-payer health insurance system and serviced by 12 ICUs containing 82 Level 1 or Level 2 adult ICU beds [11, 21].

As will be discussed in greater detail, the new normalization factor sought to include all those who developed critical illness, excluding patients who “were not candidates” for ICU admission. We conceptualized people with critical illness as belonging to one of three categories: admitted to ICUs; died without admission to an ICU; and survived without ICU care. Our data only allow identification of the first two categories. We considered persons who were not candidates for ICU admission to be those formally identified as having received palliative care. Inpatient palliative care was recognized in hospital abstracts by ICD-10-CM palliative care diagnosis codes, or codes indicating that the patient’s primary inpatient care team was the palliative care service. Outpatient palliative care was identified by specific codes in the provincial Long-Term Care and Homecare databases, or the provincial prescription drug database indicating medications supplied under a palliative care program.

We estimated the number of people ≥ 18 years old in a given year who experienced critical illness suitable for ICU care as the number who were admitted to an ICU and/or died, excluding deaths for people in palliative care within 2 years of admission. We refer to this combined group as the Potential ICU Admission Pool. To avoid double counting of individuals, we operationally calculated the Potential ICU Admission Pool as the sum of all individuals admitted to an ICU and nonpalliative deaths among people not admitted to an ICU in that year (Additional file 1: Further Explication).

We assessed yearly rates of admission for Manitoba residents to provincial ICUs over 11 years, April 1, 2004–March 31, 2015. We compared yearly rates of ICU care using three different denominators: the population, obtained from the Manitoba Health Insurance Registry, producing population-normalized rates of ICU care; the Potential ICU Admission Pool, producing critical illness-normalized rates of ICU care; and the number of people who were hospitalized for reasons other than childbirth, obtained from provincial hospital abstracts, producing nonobstetrical hospitalization-normalized rates of ICU care. Obstetrical hospitalizations were excluded from the latter because their high numbers but low association with critical illness would distort assessment of gender-related differences in ICU care. In a sensitivity analysis, we excluded elective ICU admissions.

We assessed rates of ICU care stratified by age, gender, and SES. The SES was assessed as average household income within geographic dissemination areas [22], based on the 2011 Canadian census, divided into separate quintiles for urban and rural residents, plus a small group for whom income levels could not be calculated, mainly those in chronic care facilities and other institutions.

Comparison between proportions used Fisher’s exact test. Unless otherwise indicated, comparisons were performed on data averaged over the 11 study years. Rates were age-adjusted using the direct method, with the general Manitoba population on March 31, 2015 as a reference. To make age-adjusted statistical comparisons between SES subgroups, we used grouped Poisson regression modeling of admission to ICUs with independent variables of the subgroups of interest and categorized age. Where indicated, Bonferroni adjustment was made for multiple comparisons. Analysis used SAS 9.0 (SAS Institute, Cary, NC, USA) and Stata 11.1 (Statacorp, College Station, TX, USA).

## Results

**Fig. 1:**
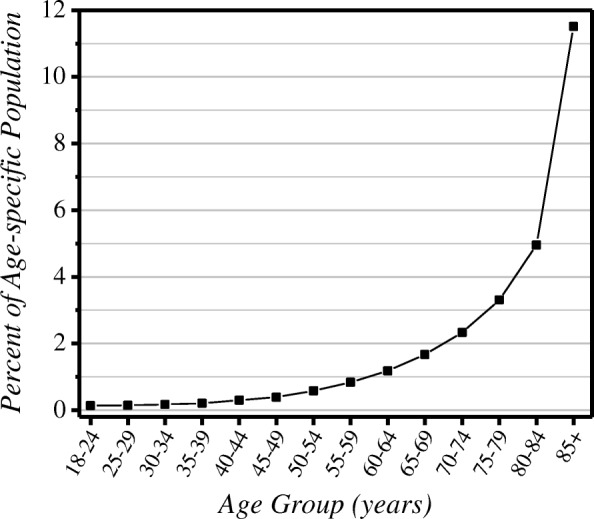
Size of the Potential ICU Admission Pool as a fraction of the population, by age. Data are unweighted averages over all 11 years

The number of distinct individuals admitted yearly to ICUs rose over the study period, from 4078 to 4491 (Table 1). Overall, 61.0% of those admitted to ICUs were men. The population rose 1.40%/year, while nonobstetrical hospitalizations declined 0.03%/year. Nonpalliative deaths fell over time, while there was a rise in individuals admitted to ICUs who did not die in that year. The Potential ICU Admission Pool generally declined slightly over the study period (Table 1). As a fraction of the population, the Potential ICU Admission Pool rose steeply with age (Fig. 1).

**Table 1:** Yearly counts of parameters used in calculating rates of ICU care, among Manitoba residents ≥ 18 years old

Year	Unique people admitted to ICUs	Population	Nonobstetrical hospitalizations	Palliative deaths,^a^ *n* (% total deaths)	Nonpalliative deaths^a^	Admitted to ICU and did not die that year	Potential ICU Admission Pool
2004/05	4078	886,141	59,179	1738 (17.6)	8140	3092	11,232
2005/06	4111	891,635	59,348	2329 (24.2)	7312	3381	10,693
2006/07	4186	897,855	58,624	3085 (31.7)	6662	3537	10,199
2007/08	4286	910,110	57,449	3075 (31.7)	6619	3587	10,206
2008/09	4241	921,181	58,427	3413 (34.7)	6411	3659	10,070
2009/10	4340	936,779	59,721	3681 (37.8)	6051	3784	9835
2010/11	4388	952,989	59,408	3796 (38.1)	6156	3870	10,026
2011/12	4401	969,591	59,558	3823 (38.7)	6062	3893	9955
2012/13	4465	988,801	58,562	4104 (41.8)	5717	4042	9759
2013/14	4443	1,005,477	57,657	4124 (41.8)	5738	4004	9742
2014/15	4491	1,018,590	58,942	4273 (40.4)	6291	4053	10,344
Unweighted average	4312	943,559	58,807	3404 (34.4)	6469	3718	10,187
Males (%)	61.0	48.8	46.2	50.7	49.0	61.6	53.6

*ICU* high intensity (Level 1 and Level 2) intensive care units in Manitoba

^a^People who died and were recorded to have been in a palliative care program within the 2 years prior to death

**Fig. 2:**
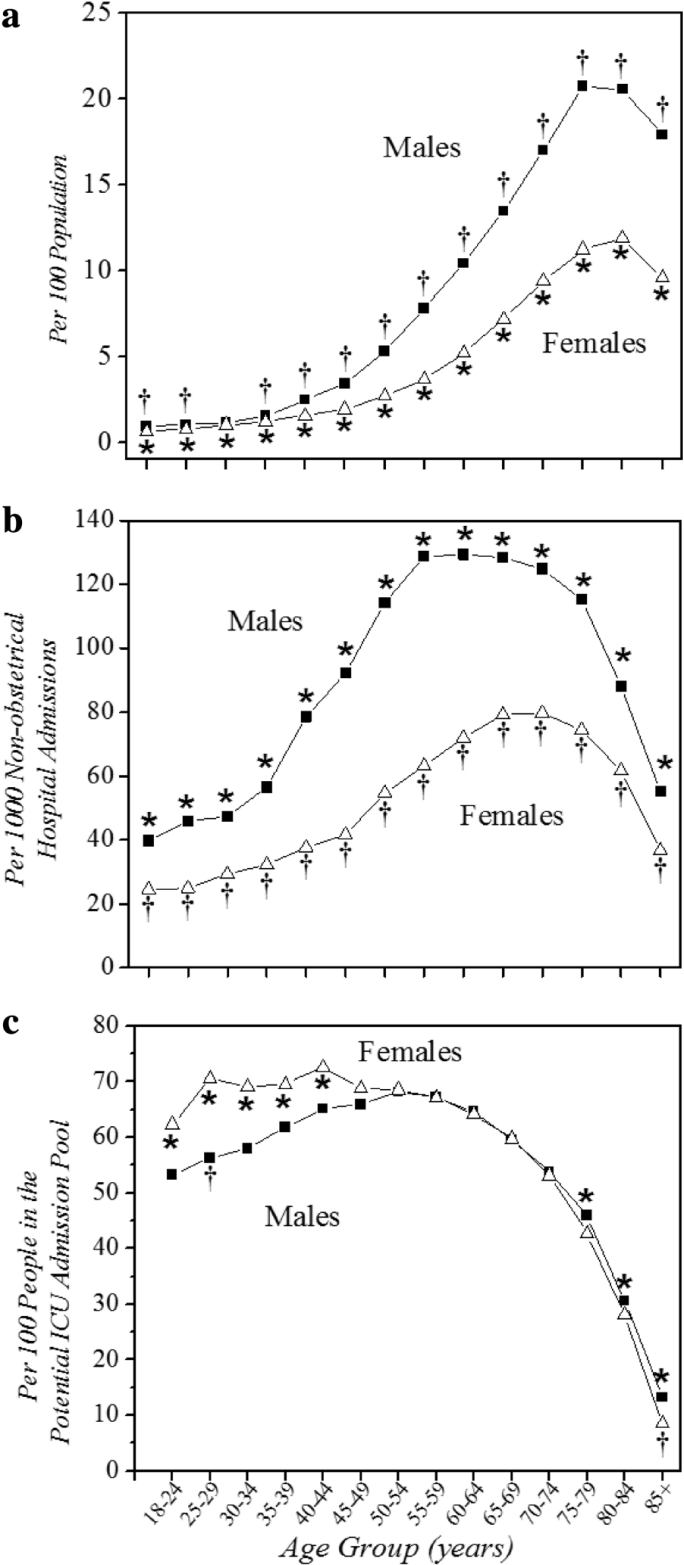
Gender-specific rates of ICU care, by age. **a** Normalized by population. **b** Normalized by nonobstetrical hospitalizations. **c** Normalized by Potential ICU Admission Pool. Data are unweighted averages over all 11 years. *p < 0.05, comparison of males to females for age category with no adjustment for multiple comparisons. †*p* < 0.05, comparison of males to females for age category with Bonferroni adjustment for 42 comparisons. ICU intensive care unit

The three different gender-specific, age-stratified rates of ICU admission show striking differences (Fig. 2, Additional file 1: Table S1). For both genders, the population-normalized rates rose steeply up to approximately age 79 years, and then plateaued or declined. Hospitalization-normalized rates showed a similar pattern, but with earlier peaks than the population-normalized rates. In contrast, critical illness-normalized rates of ICU care remained relatively constant until they began to decline after age 59 years for both genders.

**Fig. 3:**
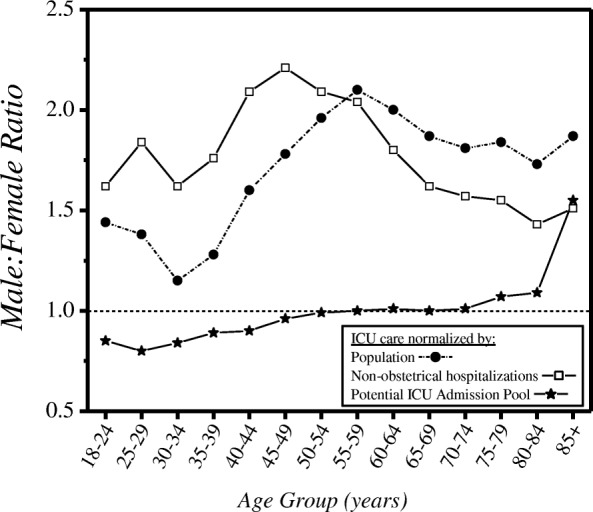
Male:female ratios of three different rates of ICU care, by age. Data are unweighted averages over all 11 years. ICU intensive care unit

**Fig. 4:**
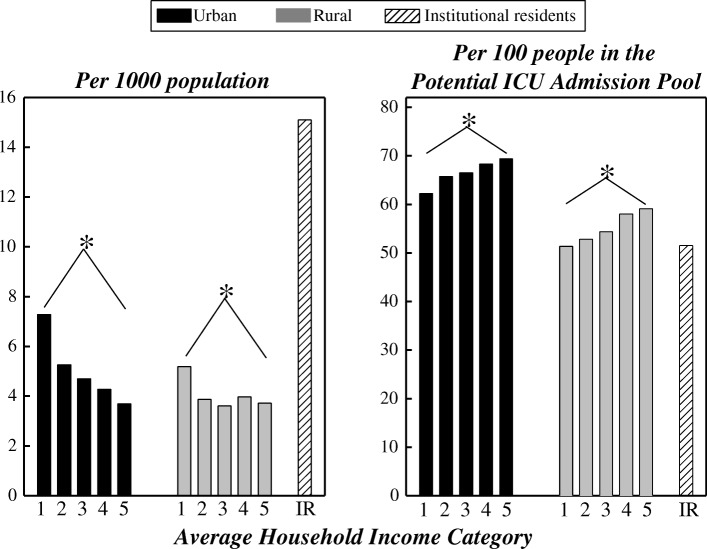
Age-adjusted rates of ICU care using two different normalizing factors, by socioeconomic status. SES increases from categories 1 to 5. Left: Normalized by population. Right: Normalized by Potential ICU Admission Pool. Bars show unweighted averages over all 11 years. *p < 0.001, comparison within indicated income quintiles. ICU intensive care unit, IR institutional residents

Male:female ratios of the different rates clarify these effects (Fig. 3). As a fraction of the population, or of hospitalized people, men had substantially higher rates than women for all age groups. However, when assessed relative to the Potential ICU Admission Pool, this excess of men mostly disappeared. With this denominator: there was a 7–14 absolute percentage excess of women in ICUs for ages 18–44 years; men and women aged 45–74 years were admitted in similar proportions; and for age ≥ 75 years, rates of ICU admission for men increasingly exceeded those for women. In absolute terms, approximately 80% of the total amount by which men outnumbered women in Manitoba ICUs occurred in the age range 45–74 years (Additional file 1: Table S2). Likewise, for analysis of ICU care by SES, the pattern was dependent on the normalizing factor used. Population-normalized rates of ICU care (Fig. 4, left panel) were higher for those with lower incomes and highest among institutionalized people. Both of these findings were reversed for critical illness-normalized rates of ICU admission (Fig. 4, right panel).

For the sensitivity analysis we excluded ICU admissions contained within elective hospital admissions. These are largely planned ICU care after certain elective surgical procedures, most commonly cardiac surgeries. This reduced the number of people admitted yearly to ICUs by 20.6%, and the Potential ICU Admission Pool by 7.4%. It did not appreciably alter the male:female ICU admission rate ratios (Additional file 1: Figure S2). This modification also did not alter the effects for SES (data not shown).

## Discussion

In this study, we developed an approximation of the incidence rate of critical illness in populations, and used it to show that across a 30-year adult age span the observed male predominance of ICU patients [7–13] was accounted for by higher rates of critical illness among men. Thus, concern that the male predominance indicates that women are disadvantaged with regard to ICU access [14] appears to be largely unfounded. Population-normalized rates are suitable for assessing healthcare utilization, but not healthcare access. For that purpose, the most appropriate denominator is the number of people who warranted such care. No prior studies of disparities in critical illness have performed such an analysis, [23] although Fransoo et al. found that higher population-normalized rates of cardiac catheterization among men are accounted for by differences in rates of underlying coronary artery disease [16].

We speculate that higher critical illness-normalized rates of ICU admission for women in younger age groups reflect the known excess among men of out-of-hospital deaths from trauma and violence [24]. Lower critical illness-normalized rates for older females could be due to a lower desire for aggressive medical care among elderly women than elderly men [25].

We computed hospitalization-based rates of ICU admission to compare with a study from British Columbia which reported that male:female ratios for hospitalization-based rates were near unity for most age groups [12]. That finding suggested that the male predominance seen in population-based rates was due to prehospital factors. However, in Manitoba the male predominance of hospitalization-based rates and population-based rates were similar overall (Fig. 3). While the reason for this difference is not clear, our data included the entire adult population of the province, while the British Columbia result derived from a single hospital.

Our second major finding was lower rates of ICU admission among critically ill persons in lower income strata. This mirrors work showing reduced access to healthcare services among the poor in developed countries, regardless of whether or not, like Canada, they provide universal, government-funded medical care [1, 26]. Analogous to studies of hospitalizations in France [27] and England [28], we found higher population-based rates of ICU admission in lower SES strata. The reversal of this gradient for critical illness-normalized rates again demonstrates the importance of choosing a denominator appropriate for assessing access to healthcare.

Differences such as these, in critical illness-normalized rates between subgroups, may result from differences in any of the following seven sequential events that define the pathway from onset of illness until ICU entry: (1) propensity toward critical illness; (2) early recognition of illness by patients; (3) willingness of patients to seek initial medical care; (4) availability of and initial access to the healthcare system, including outpatient services and hospital emergency departments; (5) admission to hospital; (6) willingness of patients for aggressive medical care as provided in ICUs; and (7) admission to the ICU. Of particular interest as regards possible gatekeeper bias is that triage decisions made by various medical gatekeepers come into play in items 4, 5, and 7. Although our analysis using critical illness-normalized rates of ICU admission is not consistent with gender-based bias in triage decisions by gatekeepers, we cannot exclude the possibility of such bias as regards SES.

Our new normalizing factor is the first to provide an estimate of the incidence of critical illness in populations [17]. While we recognize that it is only an approximation of the true underlying rate of critical illness, it nonetheless is a more appropriate denominator for assessing inequalities in ICU access than is the population count. In addition, that the male:female ratio for a 30-year age range is not only constant, but essentially unity, lends a degree of face validity to the Potential ICU Admission Pool as the appropriate normalizing factor for assessing ICU access. Prior evaluations of the incidence of various types of critical illness have used data limited to patients admitted to hospitals or ICUs, and/or with specific types of critical illness [29–34].

The main strength of our work is the reduction of potential biases by assessing an entire provincial population over 11 years. Its main limitation is that it depends on the validity of our estimate for the number of people who experienced critical illness each year and were suitable candidates for ICU admission. Each component warrants discussion and has limitations.

First, we pragmatically chose to accept that patients were critically ill if they were admitted to a high-intensity ICU. In the absence of evidence-based guidelines for what constitutes illness severe enough to warrant ICU admission [35], and with obvious differences in admission thresholds between ICUs, this choice can be criticized. However, it is challenging to devise and operationalize a better alternative.

Second is the concept that all deaths are associated with critical illness, even if that illness may have been very brief (e.g., traumatic death in the field). We justify this choice by recognizing that if such a person had been close to death at the time of discovery, rather than being dead, they might have survived long enough to be admitted to an ICU. We note that the fraction of all deaths that were palliative rose from 18 to 40% over the 11-year study interval. This may lead to concern that changing gender fractions within subcategories of death might influence our findings about the male:female ratio of ICU admissions. But in fact the male fractions of palliative deaths varied narrowly over the 11 years of the study (49.3–51.8%), as did the male fraction in ICUs (59.7–62.2%). We also note that the results were not altered in a sensitivity analysis that excluded from consideration elective ICU admissions, as commonly occur in Manitoba after elective cardiac and other high-risk surgical procedures.

Third are individuals who survived critical illness without ICU care. Although true critical illness that resolved without any medical care is likely extraordinarily rare, the number who survived with care provided outside of ICUs may not be negligible [36]. That our data lack information to estimate the size of this subset is the primary limitation of using the Potential ICU Admission Pool as a normalizing factor; however, it may be the smallest of the three main categories of critically ill people [37].

Fourth is whether and how to exclude critically ill people who “were not candidates” for ICU care. This includes critically ill people who: (i) died before they could get to an ICU; (ii) did not desire ICU care; and (iii) desired ICU care but were not accepted into the ICU by those with gatekeeping authority. While at first it seems desirable to exclude these subgroups, doing so would eliminate the ability to identify inequalities that can influence those issues, resulting in selection bias for our analysis [38]. Regarding subgroup i, economically disadvantaged persons and those in remote communities may have less access to timely care for catastrophic illness, causing higher rates of death. Subgroup ii likewise has potential for bias, as there are inequalities in access to palliative care [39]. Also, patients’ decisions to forego aggressive care are substantially influenced by their physicians [40], who can make care recommendations biased by factors such as gender without realizing it [41]. Also, as subgroup iii represents subjective, value-based decisions by gatekeepers, it is also prone to variation and bias. The only members of subgroups i–iii we can identify are those identified as having received palliative care in administrative data sources. While it would be desirable to have validated measures for all individuals who were not candidates for ICU admission by virtue of receiving symptom-focused end-of-life care, methods to do this using administrative data do not currently exist [42, 43]. What is known is that the use of ICD-9 diagnosis coding of palliative care among inpatients fails to identify many patients who actually received such care [44, 45], with such false-negatives being more common in outpatients than inpatients, and in the oldest age groups [42]. Indeed, the lower critical illness-normalized rates of ICU admission we observed in lower SES strata (Fig. 2c) could be due, in part, to underestimating decisions to limit aggressive care for those groups [39]. Thus, while pragmatic, this aspect of our calculation of the Potential ICU Admission Pool is also admittedly imperfect.

Indeed, any calculation of the number of people who experienced critical illness each year and were candidates for ICU admission would only be an approximation. Going beyond our approximation could involve the combination of: manual review of individual medical records including those pre hospital and in hospital, as well as autopsy and other death-related records for out-of-hospital deaths; and consensus definitions of what constitutes critical illness, candidates for ICU care, and avoidable deaths. Unfortunately, the latter do not exist or are poorly developed. There is not even agreement about what constitutes critical illness, as evidenced by the paucity of attempts over the past 30 years to devise a set of general ICU triage criteria [35, 46], and by the large variation, seen at multiple levels, in observed ICU admission patterns [47–51]. As for manual chart reviewing, its validity is limited, as indicated by poor agreement between reviewers [52–54]. While performing such time-consuming, labor-intensive manual reviews would render such methods incapable of use in large databases such as administrative data, manual chart reviewing would be feasible as an exercise to further validate our methods. Ultimately, we recognize that our method carries risk of unknown biases.

To assess the robustness of our findings, we conducted sensitivity analyses using modified versions of the Potential ICU Admission Pool. The first analysis used a denominator calculated as the sum of all deaths plus ICU admissions, and therefore it did not exclude deaths among those in palliative care. The second was normalized by total deaths, reasoning that this metric should track, although not equal, the number of critically ill people in the population. This latter normalization has been used before to evaluate medical resource use for critically ill people [55]. For assessing gender differences in ICU care, these two alternative denominators also dramatically reduced the male:female ratio evident in population-based rates, although to a lesser degree compared to our critical illness-normalized rates (Additional file 1: Figure S1). The similarities of findings between denominators that did and did not exclude deaths of those in palliative care provide assurance that our findings are not a result of differences in palliative care use between subgroups.

## Conclusions

We have demonstrated that most of the male predominance of ICU admission is a consequence of men having a higher rate of critical illness than women. The new factor we developed, the Potential ICU Admission Pool, was designed to allow for more appropriate analysis and comparison of ICU access. In light of the considerations detailed, we recognize that there is no unimpeachable method of estimating the number of people who should be admitted to ICUs. Accordingly, in devising this factor, we made a number of pragmatic choices, which while imperfect, provide a method for assessing ICU access that is superior to those that have been used to date. In comparison to population-normalized rates, critical illness-normalized rates led to very different conclusions about rates of ICU admission by gender and income. As this is the first effort using our new methodology, future studies are needed both for confirmation of our findings and to further explore these associations.

## Additional file

Additional file 1: Further explication of the Potential ICU Admission Pool and critical illness-normalized rates of ICU care. **Table S1.** Comparison of three different rates of ICU care, by gender and age. Data are unweighted averages over all 11 years, at the level of individuals. *p* values compare average rates for women vs men. **Table S2.** Estimated age composition of male predominance of ICU admission in Manitoba for 2010. **Figure S1.** Comparison of male:female ratios of four different rates of ICU care, by age. Data are unweighted averages over all 11 years. **Figure S2.** Comparison of male:female ratios of four different rates of ICU care, by age. Data are unweighted averages over all 11 years (DOC 130 kb)

## Abbreviations

ICD: International Classification of Diseases
ICU: Intensive care unit
SES: Socioeconomic status
