# Supplementary material for: Reassessing access to intensive care using an estimate of the population incidence of critical illness
Source: Crit Care. 2018 Aug 20;22:208. doi: 10.1186/s13054-018-2132-8 (PMC6100704; doi:10.1186/s13054-018-2132-8)
Supplement: Supplementary file 1 — Further explication of the Potential ICU Admission Pool and critical illness-normalized rates of ICU care. Table S1. Comparison of three different rates of ICU care, by gender and age. Data are unweighted averages over all 11 years, at the level of individuals. p values compare average rates for women vs men. Table S2. Estimated age composition of male predominance of ICU admission in Manitoba for 2010. Figure S1. Comparison of male:female ratios of four different rates of ICU care, by age. Data are unweighted averages over all 11 years. Figure S2. Comparison of male:female ratios of four different rates of ICU care, by age. Data are unweighted averages over all 11 years (DOC 130 kb) [file 13054_2018_2132_MOESM1_ESM.doc]

**ADDITIONAL FILE 1**

**Reassessing Access to Intensive Care Using An Estimate of the Population Incidence of Critical Illness**

Allan Garland, MD, MA, Kendiss Olafson MD, MPH, Clare D. Ramsey, MD, MSc,

Marina Yogendran, MSc, and Randall Fransoo, PhD

PAGE CONTENTS .

2 Further Explication of the Potential ICU Admission Pool and Critical illness-normalized rates of ICU care

3 Additional Table 1

4 Additional Table 1

5 Additional Figure 1

6 Additional Figure 2

Further Explication of the Potential ICU Admission Pool

In the main text we describe this cohort is those admitted to ICUs plus all nonpalliative deaths. However, it is important to avoid double counting individuals, as some people fit into both categories. The Venn Diagram below illustrates the complete situation for any given year.

- The complete blue circle (segments 1+2+3) comprises everyone admitted to an ICU in that year

- The complete orange circle (segments 2+3+4+5) comprises everyone who died in that year

- The complete white circle (segments 3+4) comprises palliative deaths. Thus segments 2+5 comprises all non-palliative deaths.

- Segment 1 (blue) = those admitted to an ICU who did not die in that year.

- Segment 2 (red) = nonpalliative deaths who were admitted to an ICU in that year.

- Segment 3 (green) = palliative deaths who were admitted to an ICU in that year.

- Segment 4 (white) = palliative deaths who were not admitted to an ICU in that year.

- Segment 5 (orange) = nonpalliative deaths who were not admitted to an ICU in that year.

1

4

5

2

3

admitted to icu all deaths palliative deaths

1+2+3 2+3+4+5 3+4

- Potential ICU Admission Pool = all those admitted to ICUs + all nonpalliative deaths *without double counting*

= all those admitted to ICUs + nonpalliative deaths not admitted to ICUs

= Entire Blue circle (Segments 1+2+3) + Orange area (Segment 5)

= Segments 1+2+3+5

- So, the critical illness-normalized rate of ICU care takes the form: A/(A+B) where:

• A=#people admitted to an ICU B=#non-palliative deaths not admitted to an ICU

• In comparing this rate between two groups, if A1/(A1+B1) = A2/(A2+B2) where A1A2 means that B1/A1=B2/A2, not that B1=B2

• So for a given age range for which the number of Men vs. Women admitted to ICUs in given year are unequal, but critical-illness normalized rates of ICU admission are the same, what is equal is this quantity:

(#nonpalliative deaths not admitted to ICU)/(# people admitted to an ICU)

not simply the number of nonpalliative deaths.

**Table S1**. Comparison of the 3 different rates of ICU care, by gender and age. Data are unweighted averages over all 11 years, at the level of individuals. p-values compare average rates for women vs. men.

| Age  Group | Population-normalized rates  (per 1000 population) | | | | Hospitalization-normalized rates  (per 100 non-obstetrical hospitalizations) | | | | Critical illness-normalized rates  (per 100 people in the  Potential ICU Admission Pool) | | | |
| --- | --- | --- | --- | --- | --- | --- | --- | --- | --- | --- | --- | --- |
|  | Male | Female | M-F  difference | p-value | Male | Female | M-F  difference | p-value | Male | Female | M-F  difference | p-value |
| 18-24 | 0.94 | 0.66 | 0.29 | <.0001 | 39.66 | 24.51 | 15.15 | <.0001 | 53.22 | 62.32 | -9.10 | 0.011 |
| 25-29 | 1.05 | 0.76 | 0.29 | <.0001 | 45.86 | 24.88 | 20.98 | <.0001 | 56.26 | 70.58 | -14.32 | 0.001 |
| 30-34 | 1.17 | 1.02 | 0.15 | 0.03 | 47.27 | 29.26 | 18.01 | <.0001 | 57.94 | 69.12 | -11.18 | 0.006 |
| 35-39 | 1.55 | 1.21 | 0.34 | <.0001 | 56.58 | 32.24 | 24.34 | <.0001 | 61.78 | 69.57 | -7.79 | 0.040 |
| 40-44 | 2.50 | 1.57 | 0.94 | <.0001 | 78.60 | 37.64 | 40.96 | <.0001 | 65.17 | 72.57 | -7.40 | 0.022 |
| 45-49 | 3.44 | 1.93 | 1.51 | <.0001 | 92.20 | 41.64 | 50.56 | <.0001 | 65.90 | 68.88 | -2.98 | 0.271 |
| 50-54 | 5.33 | 2.72 | 2.61 | <.0001 | 114.27 | 54.65 | 59.62 | <.0001 | 68.16 | 68.53 | -0.37 | 0.872 |
| 55-59 | 7.81 | 3.71 | 4.10 | <.0001 | 128.72 | 63.18 | 65.54 | <.0001 | 67.33 | 67.18 | 0.15 | 0.944 |
| 60-64 | 10.43 | 5.21 | 5.22 | <.0001 | 129.39 | 71.99 | 57.40 | <.0001 | 64.58 | 64.20 | 0.38 | 0.836 |
| 65-69 | 13.46 | 7.18 | 6.28 | <.0001 | 128.42 | 79.21 | 49.21 | <.0001 | 59.53 | 59.75 | -0.22 | 0.896 |
| 70-74 | 17.01 | 9.38 | 7.62 | <.0001 | 124.88 | 79.77 | 45.11 | <.0001 | 53.80 | 53.07 | 0.73 | 0.625 |
| 75-79 | 20.75 | 11.25 | 9.50 | <.0001 | 115.28 | 74.45 | 40.84 | <.0001 | 45.94 | 42.76 | 3.18 | 0.009 |
| 80-84 | 20.55 | 11.89 | 8.66 | <.0001 | 88.13 | 61.61 | 26.52 | <.0001 | 30.60 | 28.29 | 2.31 | 0.007 |
| 85+ | 17.93 | 9.61 | 8.32 | <.0001 | 55.16 | 36.61 | 18.55 | <.0001 | 13.21 | 8.51 | 4.70 | <.0001 |

**Table S2**. Estimated age composition of the male predominance of ICU admission in Manitoba for 2010.

| Age  group | Rates of ICU  admission  (per 1000 population)* | | Population/1000† | | estimated  # of individulas  admitted to ICUs  (rate x population) | | #males-#females  (% of total) |
| --- | --- | --- | --- | --- | --- | --- | --- |
|  | Male | Female | Male | Female | Male | Female |  |
| 18-24 | 0.94 | 0.66 | 61.8 | 60.5 | 58 | 40 | 87 (9.1%) |
| 25-29 | 1.05 | 0.76 | 40.9 | 41.1 | 43 | 31 |  |
| 30-34 | 1.17 | 1.02 | 39.0 | 39.6 | 46 | 40 |  |
| 35-39 | 1.55 | 1.21 | 39.6 | 39.7 | 61 | 48 |  |
| 40-44 | 2.50 | 1.57 | 40.8 | 40.7 | 102 | 64 |  |
| 45-49 | 3.44 | 1.93 | 47.2 | 47.2 | 162 | 91 | 770 (80.4%) |
| 50-54 | 5.33 | 2.72 | 45.2 | 44.5 | 241 | 121 |  |
| 55-59 | 7.81 | 3.71 | 38.9 | 39.8 | 304 | 148 |  |
| 60-64 | 10.43 | 5.21 | 32.9 | 33.9 | 343 | 177 |  |
| 65-69 | 13.46 | 7.18 | 23.8 | 24.9 | 320 | 178 |  |
| 70-74 | 17.01 | 9.38 | 17.6 | 19.7 | 300 | 185 |  |
| 75-79 | 20.75 | 11.25 | 14.1 | 17.4 | 293 | 196 | 101 (10.5%) |
| 80-84 | 20.55 | 11.89 | 10.3 | 15.2 | 212 | 180 |  |
| 85+ | 17.93 | 9.61 | 8.5 | 18.7 | 152 | 180 |  |
| ALL |  | | | | | | 958 (100%) |

* from Supplemental Table 1

† obtained from: Manitoba Health. Manitoba Population Report: June 1, 2010. https://www.gov.mb.ca/health/population/pr2010.pdf. Accessed January 28, 2018.

**Figure S1**. Comparison of male:female ratios of four different rates of ICU care, by age. Data are unweighted averages over all 11 years.

**Figure S2**. Comparison of male:female ratios of ICU care, by age. Data are unweighted averages over all 11 years.

Solid symbols include all ICU admissions.

Open symbols exclude elective ICU admissions, identified as being contained within elective hospital admissions.
